# Machine-learning CT radiomics for prognostication in unresectable pancreatic cancer

**DOI:** 10.3389/fphar.2026.1750762

**Published:** 2026-04-13

**Authors:** Wenqi He, Chun Cao, Qi Li, Ruoxue Yang, Xuan Yu, Wei Wang, Youqiang Hu, Ping Jiang, Min Luo

**Affiliations:** 1 Department of Radiology, Zigong Fourth People’s Hospital, Zigong, China; 2 Department of Oncology, Zigong First People’s Hospital, Zigong, China; 3 Department of Oncology, Affiliated Santai Hospital of North Sichuan Medical College, Mianyang, China; 4 The First Clinical College, Chongqing Medical University, Chongqing, China; 5 State Key Laboratory of Oncology in South China, Guangdong Key Laboratory of Nasopharyngeal Carcinoma Diagnosis and Therapy, Guangdong Provincial Clinical Research Center for Cancer, Sun Yat-sen University Cancer Center, Guangzhou, China

**Keywords:** CT, immunotherapy, machine-learning, pancreatic cancer, radiomics, radiotherapy

## Abstract

**Background:**

We aimed to develop an interpretable radiomics–clinical model to predict overall survival (OS) in unresectable pancreatic cancer (PC).

**Methods:**

In this retrospective cohort, 202 patients with unresectable PC were enrolled. A total of 1,130 radiomics features were extracted from a region of interest encompassing the largest primary lesion using 3D-Slicer. Least absolute shrinkage and selection operator (LASSO)-selected features associated with OS were used to construct a radiomics risk score (RS). Independent clinical predictors were identified through stepwise Cox regression. A nomogram integrating RS with independent clinical predictors was built.

**Results:**

Median OS (mOS) for the entire cohort was 20.3 months. From 1,130 baseline CT radiomics features, LASSO retained 12 prognostic descriptors, which were linearly combined to compute a radiomics RS. Stepwise Cox regression identified age, sex, and CA19-9 as independent clinical predictors. A nomogram integrating RS with these variables was constructed in the training set. In the validation set, the area under the receiver operating characteristic curve (AUC) reached 0.804, 0.812, and 0.794 for 1-, 2-, and 3-year OS, respectively.

**Conclusion:**

An interpretable radiomics–clinical nomogram provided accurate survival prediction in unresectable pancreatic cancer.

## Background

Pancreatic cancer (PC) remains one of the deadliest malignancies because most cases are diagnosed at an advanced stage. Recent studies have highlighted promising approaches for earlier detection, including circulating exosomal microRNAs such as hsa-let-7f-5p and CT-based radiomics strategies for predicting nodal metastasis, which may help identify high-risk patients sooner and guide treatment decisions (Sren et al., 2025; Ren et al., 2025). Nevertheless, even when a diagnosis is made, treatment of PC remains challenging. A dense desmoplastic stroma, poor perfusion, and an immunologically “cold” microenvironment impede drug delivery and dampen antitumor immunity (Mukherji et al., 2022). Marked genomic and phenotypic heterogeneity further complicate therapy, driving primary resistance and rapid relapse. Although incremental advances have been made in supportive care and systemic therapy, the overall 5-year survival rate remains dismal, underscoring an urgent need for more precise treatment strategies (Xie et al., 2024).

For patients with unresectable PC, multimodal therapy represents the standard of care (Kolbeinsson et al., 2023). Systemic chemotherapy forms the foundation, while modern radiotherapy techniques such as intensity-modulated radiotherapy (IMRT) or stereotactic body radiotherapy (SBRT) contribute to cytoreduction, palliation, and potential downstaging (Bittner et al., 2015; Burkoň et al., 2022). Despite improved local control with chemoradiation, overall survival remains limited (Versteijne et al., 2022). Recent integration of immunotherapy (particularly PD-1/PD-L1 blockade) with radiotherapy and chemotherapy—termed immuno-radio-chemotherapy (iRCT)—aims to enhance antitumor immunity through immunogenic cell death (Feng et al., 2017). However, therapeutic responses are heterogeneous, underscoring an urgent need for reliable tools to identify patients most likely to benefit from iRCT (Karamitopoulou et al., 2021; van den Ende et al., 2020).

Accurate pretreatment risk stratification is, therefore, critical as it informs key clinical decisions, including therapeutic intensification, adaptive treatment modifications, and tailored follow-up (Wang et al., 2025). Radiomics offers a noninvasive approach to quantifying tumor and peritumoral heterogeneity from routine imaging, capturing high-dimensional data that reflect underlying biology (Su et al., 2025a; Mukherjee et al., 2022). Coupled with machine learning—which enables robust feature selection, dimensionality reduction, and multimodal data integration—radiomics provides a powerful framework for developing prognostic models (Huang et al., 2022).

Building on this rationale, the present study will develop and validate integrated radiomics–machine learning models to forecast outcomes in unresectable PC treated with iRCT. We aim to identify patients most likely to benefit, refine therapeutic choices, and inform the design of prospective precision-oncology trials.

## Methods

### Study population and ethics

This retrospective, multicenter cohort study consecutively enrolled 202 patients with PC at three tertiary hospitals from January 2018 to December 2024. The protocol was approved by the Ethics Committee of Zigong Fourth People’s Hospital (EC-2025-178) and was in compliance with the Declaration of Helsinki. All patients provided written informed consent, and data were de-identified prior to analysis.

### Inclusion criteria

The inclusion criteria were as follows: 1. age ≥18 years; 2. histologically confirmed pancreatic cancer; 3. unresectable disease as determined by imaging and multidisciplinary tumor board review; and 4. imaging requirements: CT scans suitable for radiomics analysis with adequate image quality and complete acquisition parameters; 5. data completeness: available baseline clinical data, detailed treatment information, and follow-up records.

### Exclusion criteria

The exclusion criteria were as follows: 1. another active malignancy within the past 5 years; 2. non-qualifying imaging: significant motion/metal artifacts, missing slice thickness or matrix information, or incomplete contrast phases that preclude reliable segmentation or feature extraction; 3. missing key information or insufficient follow-up; and 4. pregnancy or lactation.

### CT acquisition

Contrast-enhanced CT scans were acquired using a Siemens SOMATOM Force dual-source CT scanner. Acquisition parameters were standardized across institutions, including a tube voltage of 120 kV, a tube current of 180 mAs, a rotation time of 0.25 s, a pitch of 1.7 mm, a slice thickness of 5 mm, and administration of the same iodinated contrast agent (Iohexol).

### Treatment

All patients received concurrent radio-chemotherapy, and a subset of patients additionally received immunotherapy. The gross tumor volume (GTV) was defined as the radiologically evident primary pancreatic mass and any involved regional lymph nodes. The clinical target volume (CTV) expanded upon the GTV to include regions of potential microscopic spread, including perineural invasion sites and lymphatic drainage basins, typically extending 5–10 mm around the pertinent pancreatic vasculature. A further 5–10 mm margin was added to create the planning target volume (PTV) to account for setup uncertainties.

### Radiomics feature extraction

For radiomics analysis, the portal venous phase contrast-enhanced CT images were used for region of interest (ROI) segmentation and feature extraction. The same imaging phase was consistently applied across all patients to ensure the comparability of radiomics features. ROIs were manually delineated in 3D Slicer by two senior physicians, including a professor of radiology and a professor of oncology, both with extensive experience in abdominal imaging and oncologic imaging interpretation. All segmentations were performed independently, and both physicians were blinded to clinical outcomes throughout the process. A total of 1,130 quantitative CT radiomics features were extracted from ROIs using 3D Slicer. Each ROI corresponded to the largest primary pancreatic lesion. The extracted feature set was exported for subsequent preprocessing and modeling.

### Machine learning model development

All radiomics features were standardized using z-score normalization prior to model development. We applied the least absolute shrinkage and selection operator (LASSO) regression to the 1,130 CT-derived radiomics features to select those associated with overall survival (OS). A patient-level radiomics risk score (RS) was then calculated as the weighted sum of the selected features using the corresponding LASSO coefficients. Independent clinical prognostic factors for OS were identified via stepwise Cox proportional hazards regression. All patients were randomly split into a training set and an internal validation set in a 7:3 ratio. In the training set, we constructed a nomogram by integrating the selected radiomics features with the independent clinical predictors. In the validation set, model performance was evaluated using receiver operating characteristic (ROC) analysis, decision curve analysis (DCA), and calibration.

### Statistical analysis

Baseline characteristics were summarized as the mean ± SD or median (IQR) for continuous variables and counts (percentages) for categorical variables. Normality was assessed using the Shapiro–Wilk test. Between-group comparisons used a student’s t-test or Mann–Whitney U test for continuous variables and a χ^2^ test or Fisher’s exact test for categorical variables. OS was estimated using the Kaplan–Meier method and compared between the two groups using the log-rank test. Prognostic factors were selected using a two-stage Cox approach. First, candidate variables were screened through univariate Cox regression (retention threshold: *p* < 0.05). Variables meeting this criterion were then incorporated into a multivariable Cox proportional hazards model with backward stepwise elimination (elimination criterion: *p* ≥ 0.05). The variables retained in the final stepwise model were considered independent prognostic factors. All tests were two-sided, with *p* < 0.05 considered statistically significant.

## Result

### Baseline characteristics

A total of 202 patients with PC were included and randomly assigned to a training cohort (n = 140) and a validation cohort (n = 62) in a 7:3 ratio. The overall mean age was 61.6 ± 10.6 years, and 56.9% were male. Comorbidities included diabetes mellitus in 20.3% and hypertension in 28.7%. The clinical stage was predominantly advanced: stage IV (55.0%) and stage III (25.2%), with stage I (8.91%) and stage II (10.9%). According to the T classification, T4 was the most common (44.6%), followed by T3 (17.3%), T2 (20.8%), T1 (3.96%), and Tx (13.4%). N classification was as follows: N0 44.6%, N1 33.2%, N2 7.43%, and unknown (Nx) 14.9%. Metastatic disease (M1) was present in 55.0%. The mean maximal tumor diameter was 4.22 ± 1.82. Laboratory indices at baseline included WBC 6.18 ± 3.27 × 10^9^/L, neutrophils 4.21 ± 3.58 × 10^9^/L, total bilirubin 39.9 ± 71.4 μmol/L, and CA19-9 1,179 ± 2472 U/mL. There were no significant differences between the training and validation cohorts across these baseline variables (all *p* > 0.05), indicating well-balanced groups (Table 1).

**TABLE 1:** Baseline characteristics of the cohort.

Patients	All	Train	Test	*p*
Variable	202	140	62	​
Age, years (mean ± SD)	61.6 (10.6)	61.8 (10.8)	61.2 (10.3)	0.685
Sex	​	​	​	0.242
Female	87 (43.1%)	56 (40.0%)	31 (50.0%)	​
Male	115 (56.9%)	84 (60.0%)	31 (50.0%)	​
Diabetes mellitus	​	​	​	0.242
No	161 (79.7%)	108 (77.1%)	53 (85.5%)	​
Yes	41 (20.3%)	32 (22.9%)	9 (14.5%)	​
Hypertension	​	​	​	0.438
No	144 (71.3%)	97 (69.3%)	47 (75.8%)	​
Yes	58 (28.7%)	43 (30.7%)	15 (24.2%)	​
Stage	​	​	​	0.862
I	18 (8.91%)	13 (9.29%)	5 (8.06%)	​
II	22 (10.9%)	16 (11.4%)	6 (9.68%)	​
III	51 (25.2%)	33 (23.6%)	18 (29.0%)	​
IV	111 (55.0%)	78 (55.7%)	33 (53.2%)	​
T	​	​	​	0.487
T1	8 (3.96%)	5 (3.57%)	3 (4.84%)	​
T2	42 (20.8%)	31 (22.1%)	11 (17.7%)	​
T3	35 (17.3%)	20 (14.3%)	15 (24.2%)	​
T4	90 (44.6%)	64 (45.7%)	26 (41.9%)	​
Tx	27 (13.4%)	20 (14.3%)	7 (11.3%)	​
N	​	​	​	0.523
N0	90 (44.6%)	59 (42.1%)	31 (50.0%)	​
N1	67 (33.2%)	47 (33.6%)	20 (32.3%)	​
N2	15 (7.43%)	10 (7.14%)	5 (8.06%)	​
Nx	30 (14.9%)	24 (17.1%)	6 (9.68%)	​
M	​	​	​	0.861
M0	91 (45.0%)	62 (44.3%)	29 (46.8%)	​
M1	111 (55.0%)	78 (55.7%)	33 (53.2%)	​
Tumor size, cm, (mean ± SD)	4.22 (1.82)	4.19 (1.83)	4.30 (1.82)	0.709
WBC, ×10^9^/L, (mean ± SD)	6.18 (3.27)	6.12 (2.56)	6.30 (4.51)	0.761
Neutrophils, ×10^9^/L, (mean ± SD)	4.21 (3.58)	4.23 (3.36)	4.18 (4.05)	0.937
TBIL, μmol/L, (mean ± SD)	39.9 (71.4)	41.5 (72.0)	36.4 (70.5)	0.641
CA19-9, U/mL, (mean ± SD)	1,179 (2,472)	1,179 (2,437)	1,179 (2,568)	0.999

WBC, white blood cell count; TBIL, total bilirubin; CA19-9, carbohydrate antigen 19-9.

### Survival

**FIGURE 1:**
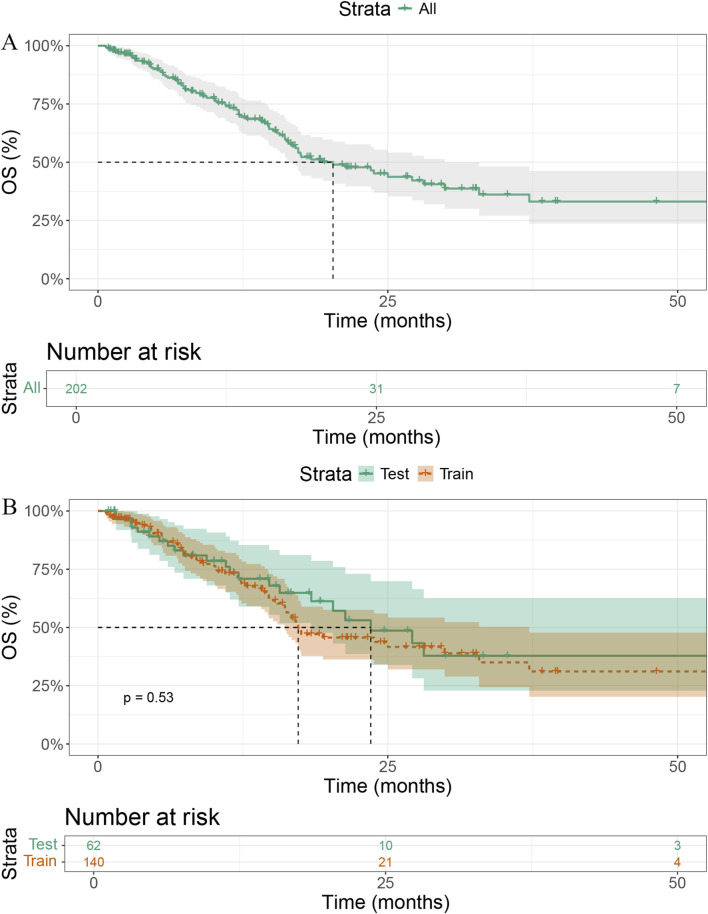
Kaplan – Meier survival analysis. **(A)** Overall survival (OS) curve of all patients (n = 202). **(B)** Comparison of OS between training (n = 140) and test (n = 62) cohorts (*p* = 0.53).

The median OS (mOS) for the entire cohort was 20.3 months (95% CI = 16.7–29.9, Figure 1A). The mOS was 17.3 months (95% CI = 16.1–37.2) in the training cohort and 23.5 months (95% CI = 18.4–NE) in the validation cohort (*p* = 0.54, Figure 1B).

### Feature selection and risk score

**FIGURE 2:**
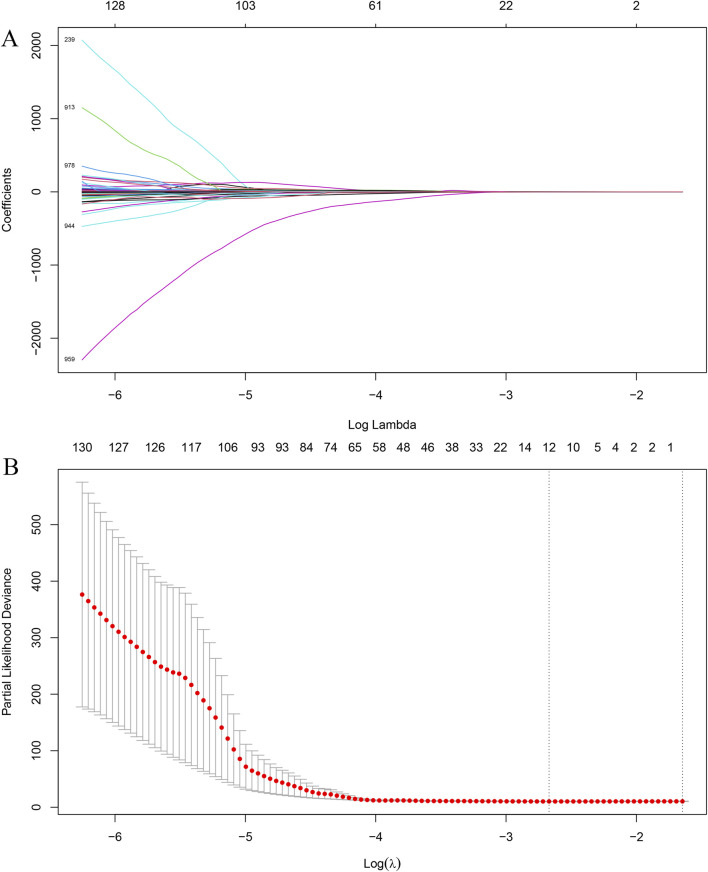
LASSO regression for feature selection. **(A)** LASSO coefficient profiles of radiomics features plotted against log(λ). **(B)** Ten-fold cross-validation for selecting the optimal λ value; the dotted line indicates the λ corresponding to the minimum partial likelihood deviance.

Using LASSO regression (Figures 2A,B) with cross-validation, we reduced the initial set of 1,130 CT-based radiomics features to 12 features associated with OS:

1. wavelet.LHL.glszm.SizeZoneNonUniformityNormalized
2. wavelet.HLH.glszm.GrayLevelNonUniformity
3. wavelet.HHH.gldm.LowGrayLevelEmphasis
4. wavelet.HHH.glszm.SmallAreaEmphasis
5. log.sigma.3.0.mm.3D.glcm.Imc1
6. log.sigma.3.0.mm.3D.gldm.LargeDependenceEmphasis
7. log.sigma.5.0.mm.3D.glcm.MaximumProbability
8. log.sigma.5.0.mm.3D.glrlm.GrayLevelNonUniformityNormalized
9. log.sigma.5.0.mm.3D.glrlm.LongRunLowGrayLevelEmphasis
10. log.sigma.5.0.mm.3D.glszm.SmallAreaLowGrayLevelEmphasis
11. wavelet.LLH.firstorder.10Percentile
12. wavelet.HHH.glszm.SizeZoneNonUniformityNormalized.

A patient-level RS was then computed as the linear combination of these features weighted using their LASSO coefficients. Independent clinical prognostic factors for OS identified through stepwise Cox proportional hazards regression were age, sex, and CA19-9 (Table 2).

**TABLE 2:** Univariate and multivariate Cox regression analysis of prognostic factors.

Variable	All	Events	HR (univariable)	HR (multivariable)	HR (final)
Age (mean ± SD)	61.6 ± 10.6	81	1.03 (1.01 – 1.06, *p* = 0.004)	1.04 (1.02 – 1.07, *p* < 0.001)	1.04 (1.02 – 1.07, *p* < 0.001)
Sex					
Female	87 (43.1%)	41	Reference	​	​
Male	115 (56.9%)	40	0.58 (0.37 – 0.90, *p* = 0.014)	0.57 (0.35 – 0.91, *p* = 0.018)	0.54 (0.34 – 0.84, *p* = 0.007)
Diabetes mellitus					
No	161 (79.7%)	65	Reference	​	​
Yes	41 (20.3%)	16	0.91 (0.53 – 1.58, *p* = 0.746)	​	​
Hypertension					
No	144 (71.3%)	58	Reference	​	​
Yes	58 (28.7%)	23	1.37 (0.84 – 2.24, *p* = 0.209)	​	​
Stage					
I	18 (8.9%)	7	Reference	​	​
II	22 (10.9%)	7	1.04 (0.36 – 2.97, *p* = 0.941)	1.21 (0.34 – 4.29, *p* = 0.764)	​
III	51 (25.2%)	22	1.90 (0.81 – 4.45, *p* = 0.142)	1.92 (0.56 – 6.60, *p* = 0.302)	​
IV	111 (55.0%)	45	2.48 (1.11 – 5.53, *p* = 0.026)	2.08 (0.70 – 6.18, *p* = 0.189)	​
T category					
T1	8 (4.0%)	2	Reference	​	​
T2	42 (20.8%)	16	2.96 (0.68 – 12.92, *p* = 0.149)	2.44 (0.54 – 11.14, *p* = 0.248)	2.37 (0.54 – 10.42, *p* = 0.254)
T3	35 (17.3%)	13	2.70 (0.61 – 12.00, *p* = 0.192)	1.52 (0.33 – 7.09, *p* = 0.591)	1.77 (0.39 – 8.08, *p* = 0.462)
T4	90 (44.6%)	38	3.53 (0.85 – 14.71, *p* = 0.083)	1.65 (0.35 – 7.82, *p* = 0.529)	2.32 (0.55 – 9.80, *p* = 0.253)
Tx	27 (13.4%)	12	5.89 (1.31 – 26.55, *p* = 0.021)	3.87 (0.75 – 19.81, *p* = 0.105)	6.11 (1.30 – 28.66, *p* = 0.022)
N category					
N0	90 (44.6%)	36	Reference	​	​
N1	67 (33.2%)	28	1.17 (0.71 – 1.91, *p* = 0.544)	1.21 (0.70 – 2.09, *p* = 0.492)	1.30 (0.78 – 2.18, *p* = 0.316)
N2	15 (7.4%)	4	0.96 (0.34 – 2.69, *p* = 0.933)	1.36 (0.43 – 4.32, *p* = 0.602)	1.59 (0.53 – 4.78, *p* = 0.405)
Nx	30 (14.9%)	13	2.18 (1.15 – 4.16, *p* = 0.017)	2.13 (1.04 – 4.35, *p* = 0.038)	2.62 (1.32 – 5.20, *p* = 0.006)
M category					
M0	91 (45.0%)	36	Reference	​	​
M1	111 (55.0%)	45	1.74 (1.12 – 2.71, *p* = 0.014)	1.12 (0.95 – 1.22, *p* = 0.522)	​
Size	4.2 ± 1.8	81	1.13 (1.02 – 1.24, *p* = 0.019)	1.05 (0.93 – 1.18, *p* = 0.438)	​
WBC	6.2 ± 3.3	81	0.99 (0.93 – 1.06, *p* = 0.764)	​	​
Neutrophils	4.2 ± 3.6	81	0.96 (0.89 – 1.05, *p* = 0.389)	​	​
TBIL	39.9 ± 71.4	81	1.00 (1.00 – 1.00, *p* = 0.260)	​	​
CA199	1,179.1 ± 2,471.5	81	1.00 (1.00 – 1.00, *p* = 0.005)	1.00 (1.00 – 1.00, *p* = 0.011)	1.00 (1.00 – 1.00, *p* = 0.002)

WBC, white blood cell count; TBIL, total bilirubin; CA19-9, carbohydrate antigen 19-9.

### Model construction and validation

**FIGURE 3:**
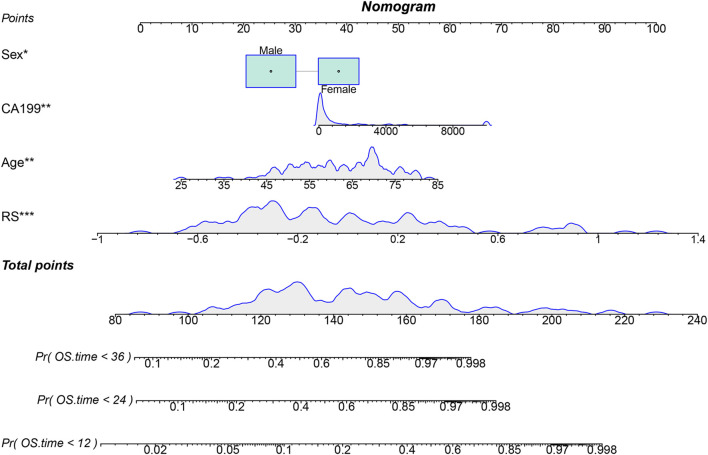
Nomogram for predicting overall survival (OS). The nomogram integrates sex, CA19-9 level, age, and radiomics score (RS) to predict 12-, 24-, and 36-month OS probabilities in patients with unresectable pancreatic cancer.

**FIGURE 4:**
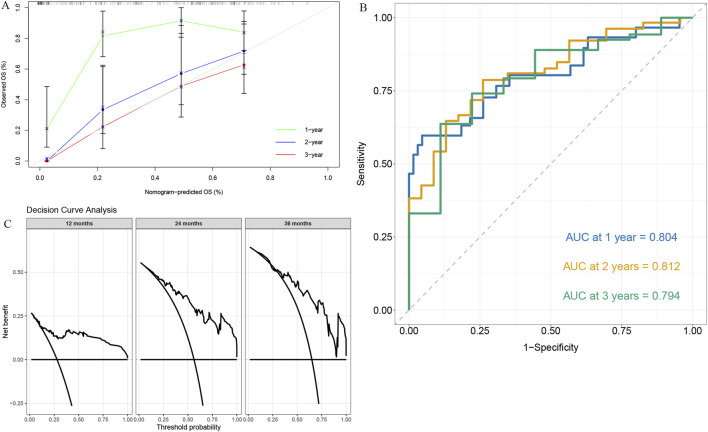
Evaluation of the nomogram performance. **(A)** Calibration curves for 1-, 2-, and 3-year OS showing good agreement between predicted and observed outcomes. **(B)** Time-dependent ROC curves of the nomogram for predicting 1-, 2-, and 3-year OS (AUC = 0.804, 0.812, and 0.794, respectively). **(C)** Decision curve analysis (DCA) demonstrating the clinical net benefit of the nomogram at 12, 24, and 36 months.

In the training cohort, we built a multivariable nomogram by integrating the RS with age, sex, and CA19-9 (Figure 3). In the internal validation cohort, calibration plots showed close agreement between predicted and observed survival (Figure 4A). Time-dependent ROC analysis yielded AUC values of 0.804, 0.812, and 0.794 for predicting 1-, 2-, and 3-year OS, respectively (Figure 4B). For the prediction of 1- and 2-year survival, the model provided a higher net benefit than the “treat-all” or “treat-none” strategies across a threshold probability range of approximately 10%–50%. For 3-year survival prediction, this superior net benefit was observed from a threshold of 30% up to 50% (Figure 4C). The Brier scores for 1-, 2-, and 3-year predictions were 0.154, 0.149, and 0.161, respectively.

## Discussion

In this study, we present an interpretable, clinic-ready nomogram that integrates a CT-based radiomics RS with routinely available clinical variables to predict overall survival in patients with unresectable pancreatic cancer. The resulting model achieved time-dependent AUC values of 0.804–0.812 in the validation set, with good calibration and a favorable net benefit on decision-curve analysis, demonstrating robust predictive performance that can inform individualized therapeutic decision-making.

PC retains an “immune-cold” phenotype characterized by dense stroma and poor immune infiltration (Morrison et al., 2018; Jenkins et al., 2025). Although radiotherapy can promote immunogenic cell death and enhance antigen presentation, clinical responses remain heterogeneous (Xu et al., 2025). Unselective application of iRCT risks unnecessary toxicity and may lead to missed alternative treatments. A pretreatment risk-stratification model is, therefore, needed to (i) select patients most likely to benefit from iRCT, (ii) identify high-risk cases for treatment intensification, and (iii) inform shared decision-making when therapeutic options are equipoised (Stoop et al., 2024).

In our model, age and sex emerged as independent predictors of overall survival. Although statistically robust, these variables should be interpreted cautiously. Neither factor is a direct tumor-intrinsic biomarker, and their prognostic effects in advanced PC are likely mediated through indirect pathways—such as comorbidity burden, treatment tolerance, immune competence, or pharmacokinetic differences—rather than reflecting underlying tumor biology. Moreover, because our dataset lacks granular measures of frailty, immune status, and treatment-related toxicities, age and sex may partly represent proxies for unmeasured confounding (Zabransky et al., 2024; Zhong et al., 2022). Therefore, their inclusion enhances pragmatic clinical risk stratification but should not be viewed as evidence for a mechanistic association. Furthermore, CA19-9, a recognized surrogate of tumor burden and aggressiveness, provided additional prognostic value (Engle et al., 2019). Integrating these readily available variables with the radiomics score creates a practical, multidimensional risk-assessment tool. The use of LASSO regression with cross-validation ensured a parsimonious and stable feature set, mitigating overfitting in this high-dimensional analysis (Li et al., 2025).

Potential clinical applications are multifold. First, the nomogram enables pretreatment risk stratification to guide initial therapeutic selection. Specifically, patients identified as low-risk can be confidently directed toward standard iRCT, whereas high-risk patients may benefit from alternative strategies such as systemic chemotherapy-dominant regimens or enrollment in trials evaluating novel therapeutic combinations. Second, surveillance intensity can be individualized according to risk: high-risk patients may merit shorter imaging intervals and earlier assessment for treatment modification, while low-risk patients could be followed less frequently, reducing unnecessary healthcare encounters. Third, the nomogram offers an objective, reproducible baseline metric for multidisciplinary tumor boards against which the potential value of experimental regimens (e.g., intensified chemotherapy backbones or molecularly targeted agents) can be rationally evaluated. Given that all required inputs—baseline contrast-enhanced CT, age, sex, and CA19-9—are routinely available in clinical practice, the model is readily deployable in real-world settings and can be reapplied at restaging timepoints to inform adaptive therapeutic strategies (Anderson et al., 2021; Shah et al., 2024; Ren et al., 2022; Cui et al., 2019).

Clinically, the nomogram can support risk stratification before iRCT. Patients classified as high-risk may benefit from earlier multidisciplinary reassessment, consideration of treatment optimization/intensification (including trial enrollment when available), and closer surveillance with earlier restaging to enable timely treatment modification. In contrast, low-risk patients may proceed with standard iRCT and routine follow-up, while intermediate-risk patients can be discussed in MDT settings when management is equipoised. The model is intended as a decision-support tool, and prospective validation is needed to define optimal risk thresholds for escalation (Su et al., 2025b).

Several limitations should be noted. First, as a retrospective study, selection bias is unavoidable, and spatiotemporal variation in iRCT regimens (differences across centers and over time) may introduce residual confounding and affect the stability and generalizability of the observed associations, despite consistent inclusion criteria across the three hospitals. Second, our handling of the serum CA19-9 biomarker has limitations; it was treated as a continuous variable without addressing extreme outliers, which may affect the model’s robustness and generalizability. Third, there was inherent clinical heterogeneity in the multimodal treatment received by patients (e.g., variations in radiotherapy techniques [IMRT vs. SBRT] and differing regimens of chemotherapy and immunotherapy). We did not perform subgroup analyses or statistical adjustments for these specific treatment modalities, which could confound the prognostic associations identified by the model. Fourth, we aimed to develop a prognostic tool for the general unresectable pancreatic cancer population. Due to the limited cohort size, we did not perform stage-stratified analysis as it would compromise the statistical power for model development. Future research with larger samples is needed to validate the model’s applicability within specific stage subgroups. In addition, the wide 95% CIs for some covariates (e.g., Tx) likely reflect sparse data, including small numbers of patients/events in that subgroup and missingness, leading to unstable HR estimates; thus, these results should be interpreted cautiously and require validation in larger cohorts. Finally, although LASSO reduces overfitting, the model is still limited by the number of events. Future studies should set appropriate events-per-variable thresholds and consider harmonization methods to reduce batch effects.

In summary, this interpretable radiomics–clinical model demonstrates robust predictive performance for overall survival in patients with unresectable pancreatic cancer treated with iRCT.

## Data Availability

The raw data supporting the conclusions of this article will be made available by the authors, without undue reservation.
